# Large-scale Implementation of Fascia Iliaca Compartment Blocks in an Emergency Department

**DOI:** 10.5811/westjem.58793

**Published:** 2023-05-03

**Authors:** Tony Downs, Joshua Jacquet, Jno Disch, Nicholas Kolodychuk, Lance Talmage, Jessica Krizo, Erin L Simon, Anita Meehan, Robert Stenberg

**Affiliations:** * Cleveland Clinic Akron General, Department of Emergency Medicine, Akron, Ohio; † Cleveland Clinic Akron General, Department of Orthopedic Surgery, Akron, Ohio; ‡ Cleveland Clinic Akron General, Department of Anesthesia, Akron, Ohio; § Cleveland Clinic Akron General, Department of Health Sciences, Akron, Ohio; || Cleveland Clinic Akron General, Department of Nursing, Akron, Ohio

## Abstract

**Introduction:**

A robust body of literature supports the use of fascia iliaca compartment blocks (FICB) for improving outcomes in hip fractures, especially in the geriatric population. Our objective in this project was to implement consistent pre-surgical, emergency department (ED) FICB for hip fracture patients and to address barriers to implementation.

**Methods:**

With the support of a multidisciplinary team, including orthopedic surgery and anesthesia, a core team of emergency physicians developed and implemented a departmentwide FICB training and credentialing program. The goal was to have 80% of all emergency physicians credentialed to provide pre-surgical FICB to all hip fracture patients seen in the ED who met the criteria. Following implementation, we assessed approximately one year of data on hip fracture patients presenting to the ED. We evaluated whether or not they were eligible for FICB and, if so, whether or not they received it.

**Results:**

Emergency physician education has resulted in 86% of clinicians credentialed to perform FICB. Of 486 patients presenting for hip fracture, 295 (61%) were considered eligible for a block. Of those eligible, (54%) consented and underwent a FICB in the ED.

**Conclusion:**

A collaborative, multidisciplinary effort is vital for success. The primary barrier to achieving a higher percentage of eligible patients receiving blocks was the deficit of emergency physicians initially credentialed. Continuing education is ongoing, including credentialing and early identification of patients eligible for the fascia iliaca compartment block.

## INTRODUCTION

Approximately 340,000 patients with hip fractures present to the emergency department (ED) each year.^1^ These occur most commonly among patients greater than 60 years of age. One challenge emergency physicians (EP) face in caring for these patients is adequate pain control. Frequently, parenteral opiates are used as the first line of pain management for hip fractures. However, the elderly population is susceptible to the adverse effects of these medications.^2^ These side effects include sedation, dizziness, delirium, constipation, and respiratory depression. The use of non-steroidal anti-inflammatory drugs also has deleterious effects. They can increase the risk of gastrointestinal bleeding and exacerbate a patient’s existing renal dysfunction. In addition, the use of opioids alone is often ineffective and leads to inadequate pain management that can place the patient at a higher risk of delirium.^3^

Regional anesthesia is an effective way of controlling pain associated with hip fractures.^2^, ^3^ The fascia iliaca compartment block (FICB) has been well studied by anesthesiologists for perioperative pain relief.^4^ The use of ultrasound-guided FICB has been associated with reduced pain scores, shorter length of stay, lower incidence of pneumonia, and fewer opiate requirements.^5^, ^6^ Although there are many documented benefits of performing FICBs preoperatively in the ED, it can be challenging to implement this procedure into an EP’s practice. One institution used a multidisciplinary initiative to train EPs with lectures, online video-narrated instructions, and hands-on sessions. The hands-on sessions involved three stations focusing on visualizing anatomy using a human model and needle utilization on models and simulators. A geriatric order set for the electronic health record was also created. Despite this implementation, the study showed that just two of 77 (2.5%) eligible patients received the FICB.^7^

Our emergency medicine residency program at an urban, Level 1 trauma center, tertiary care hospital recently implemented FICBs into our general practice. In this study our aim was to describe the implementation of a multidisciplinary initiative to credential EPs, describe the resources used for this process and the outcomes, and to identify barriers to implementation.

## METHODS

### Intended Patient Demographics

Patients included were adults who presented to the ED with femoral neck fractures, intertrochanteric fractures, or femoral shaft fractures from January 1–December 17, 2020. Patients were excluded if they had infection over the site, prior vascular surgery to the inguinal region, allergy to the anesthetic, clinical signs of femoral nerve injury or vascular injury, open fracture, polytrauma (per clinician discretion), or were on anticoagulants or antiplatelets such as warfarin (with international normalized ratio >1.4), ticagrelor, apixaban, rivaroxaban, dabigatran, and clopidogrel.

### Approval Process

Emergency physicians must be credentialed to perform nerve blocks. This process was implemented and is standard of care for patients with hip fractures. Therefore, no institutional research board approval for patient enrollment was required. Prior to implementation, while there was a rare block performed by ultrasound faculty, there was no consistent use of this procedure. The ED team consulted the institution’s ethics committee to determine how to obtain consent from patients who were unable to consent to the procedure and when challenges arose in obtaining consent while patients were in the ED. The institution’s ethics committee and ED leadership determined that for patients unable to consent to the FICB procedure, attempts would be made and documented to reach out to the patient’s healthcare power of attorney. If unable to consent, the block would be deemed emergent for this time-sensitive procedure.

**Population Health Research Capsule**

What do we already know about this issue?

*Fascia iliac compartment blocks (FICB) are a safe procedure within the skillset of emergency physicians to improve clinical outcomes*.

What was the research question?

*What methods can be used to optimize a large-scale implementation of FICBs for patients with hip fractures?*

What was the major finding of the study?

*Of eligible patients, 54% received a FICB. At end of the study period, 86% of emergency physicians were credentialed*.

How does this improve population health?

*Expanding access to FICBs allows more patients to experience the benefits including reduced pain, shorter length of stay, and theoretical decrease in delirium*.

### Implementation

**Figure 1:**
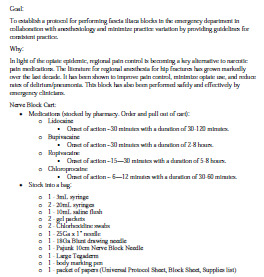
Initial fascia iliaca compartment block implementation proposal prepared for administration. *IV*, intravenous; *NS*, normal saline.

**Figure 2:**
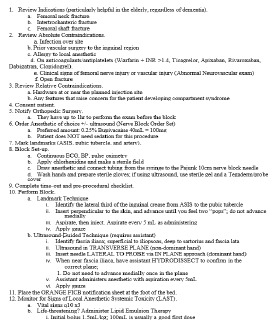
One-page reference guide for fascia iliaca compartment block procedures. INR, international normalized ratio; hr, hour; mL, milliliter; mg, milligram.

Initially, a core group of EPs (three ultrasound faculty and one vice chair) and an anesthesiologist served as the team for implementing the FICB in the ED. A hospital-wide multidisciplinary group was also created to evaluate the care of patients with hip fractures. A proposal was drafted (Figure 1), and then a FICB protocol was developed and summarized into a one-page document that functioned as a reference guide for the procedure (Figure 2).

**Figure 3:**
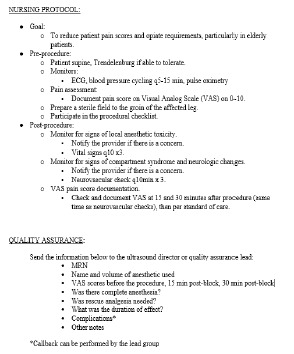
Protocol participation in fascia iliaca compartment blocks for nursing staff. *ECG*, electrocardiogram; *MRN*, medical record number.

**Figure 4:**
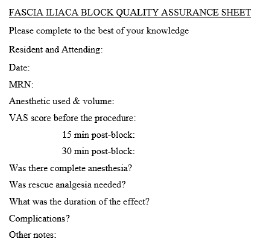
Documentation of fascia iliaca compartment block information to be submitted for continued quality assurance. *MRN*, medical record number; *VAS*, visual analog scale

**Figure 5:**
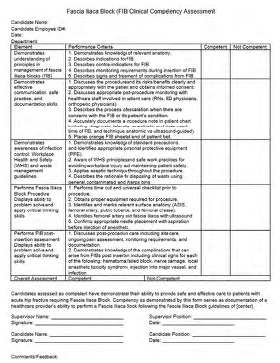
Process for documentation of clinical competency for emergency medicine providers.

**Figure 6:**
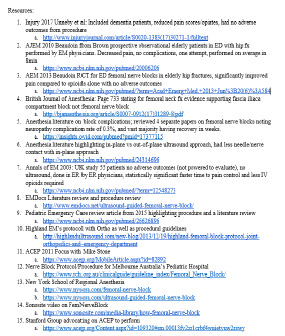
Resources used to develop fascia iliac block protocol in the emergency department.

**Figure 7:**
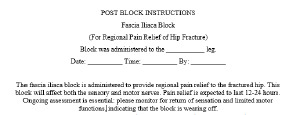
Instructions to be left on the bed of the patient following the block.

Once the protocol was in place, representatives from the other departments (orthopedic surgery, anesthesia, and nursing) formed an expert panel to develop a consensus on how the block would be implemented and what guidelines would be instituted. Nursing protocols (Figure 3), as well as documents for quality assurance (Figure 4) and assessing clinical competency (Figure 5), were drafted. References (Figure 6) and post-block instruction (Figure 7) are also provided.

### Addressing Barriers to Implementation

Measures were taken to minimize barriers to implementation of the FICB, encourage use, and prevent delays in care. These included protocols coordinated with ED pharmacists to ensure anesthetic (40 milliliters [mL] 0.25% bupivacaine) would be stocked. Information technology (IT) developed an order set for EPs. The ED nursing leadership assembled kits in the ED, which included chlorhexidine swabs, a nerve block needle, a large sterile transparent dressing, an 18-gauge drawing needle, two 20-mL syringes for drawing anesthetic, a colored, post-block instruction sheet to remain on the patient’s bed (Figure 7), and a body-marking pen. The ED also purchased 22-gauge 50 millimeter (mm) and 100 mm SonoPlex II Facet nerve block needles (Pajun GMbH Mediziatechnologie, Geisengen, Germany). The Department of Anesthesia was already using these needles for FICBs. These needles provide documented improvement of visualization under ultrasound and have a better safety profile around neurovascular structures.^8^

There is also the challenge of physicians who did not train with ultrasound feeling uncomfortable with the ultrasound-based approach, identifying the fascia iliaca and then subsequently performing an in-plane approach. One of the means used to mitigate this was including the landmark-based approach. This provides a faster set-up, is more manageable with just one person and, as implied, does not use ultrasound.

### Training and Procedural Competence

Procedural competence was determined using expert consensus by an anesthesiologist, the ED chair, the emergency ultrasound director, and the assistant emergency ultrasound director to be adequate after five successful supervised FICBs with a minimum of two live FICBs (allowing for ≤3 FICBs on a simulator). These were tracked and documented on the FICB competency worksheet. Initially, the core group of EPs became credentialed by performing blocks with anesthesia in the post-anesthesia care unit.

A program consisting of didactics (live or online lecture), a review of the ED FICB protocol, and a website were developed for training. The FICB simulator, a Simulab Regional Anesthesia Femoral Training Package, (Simulab Corporation, Seattle, WA) was purchased to implement the FICB training program and develop competency. The simulator was securely stored in the ED to be easily accessed while physicians were on shift. Due to the complexity of scheduling and coronavirus 2019, no formal, in-person course was done.

All staff EPs were provided the opportunity to become credentialed in performing FICBs, and their successful blocks were signed off by the four credentialed EPs or the anesthesiologist. Once a staff physician was credentialed, they could supervise other physicians (employing the “teach the teacher” model) and sign off on performances of a successful supervised FICB. All levels of emergency resident physicians were trained in this procedure and were able to perform a FICB under the direct supervision of a credentialed attending EP. In addition, in conjunction with the departments of anesthesia and orthopedic surgery, a core group of orthopedic surgery resident physicians were also credentialed at the same time to enhance the number of patients receiving FICB in the ED.

Throughout the process, there was encouragement by the initial core group of EPs to increase the number of credentialed clinicians performing the procedure. This was done through educational spaced repetition (an educational method to improve retention that uses a repeated review of content at different time intervals) at weekly emergency medicine conference, through access to online and written training materials, and through email communication detailing where staff physicians were in the credentialing process.^13^ Departmental statistics on the percentage of eligible patients who received the nerve block were also communicated to EPs.

### Chart Review, Data Collection, and Data Analysis

The institutional research review board approved a review of patient charts and granted a waiver of consent due to its minimal risk. Data on all patients who presented to the ED and were diagnosed with a hip fracture (as defined above) were collected as part of a QI program within the institution and used for this evaluation. Data abstracted included the date of presentation, whether the patient received a block in the ED, and whether the patient had a contraindication for a FICB. We used descriptive statistics to determine the percentages of patients eligible for and receiving the block.

## RESULTS

From January 1–December 17, 2020, 485 patients in the ED had a femoral neck fracture, intertrochanteric fracture, or femoral shaft fracture confirmed with radiographic imaging. Of the patients who presented to the ED, 295 (61%) had no contraindications to receiving a FICB. Compartment blocks were performed on 160/ 295 (54%) eligible patients after obtaining written informed consent. A total of 37 EPs (86%) are certified to perform the FICBs in the ED. These physicians can perform FICBs independently and supervise other physicians performing FICBs in the ED. During the early phases of implementation, the blocks were also completed by orthopedic surgeons consulted to the ED.

## DISCUSSION

Numerous studies have shown FICB to be a safe and effective means of pain reduction in patients with hip fractures.^6^,^9^,^10^ Anesthesia and orthopedic surgery have traditionally administered these blocks during the perioperative period. However, EPs can perform the FICB after a standardized training program.^9^ Challenges arise with the implementation of new procedures in a hospital system, and barriers to changes in patient management can occur.^11^

We used an interdisciplinary collaboration between anesthesia, orthopedic surgery, nursing, pharmacy, and IT to assist in implementing this procedure. Most EDs have not employed this type of collaboration regarding ultrasound-guided regional anesthesia.^11^ We found collaboration was fundamental for credentialing physicians caring for patients with hip fractures in the ED and optimizing the number of patients receiving blocks.

Initially, we had a goal of credentialing 95% of EPs to perform the block, but we did not reach this percentage within this timeframe. Other institutions have reported this challenge.^11^ Increasing the number of credentialed clinicians likely increases the number of blocks provided to eligible patients in the ED. There are multiple considerations on how to improve the number of credentialed clinicians: incentivizing (including monetarily) physicians to become credentialed and perform the FICBs, making credentialed clinicians available 24/7 to supervise and sign off on uncredentialed clinicians, paying physicians to come in off-shift for training, developing a hospital-wide nerve block team, developing a hospital-approved video teleconference for supervision, and requiring all physicians to become credentialed.^12^ One of the most effective pieces for credentialing was having the simulator easily accessible in the ED to help increase comfort in the moment and sign physicians off when time was found in the department or at the end of shift.

There are significant opportunities for future research. There remain opportunities to optimize the most efficient and cost-effective means to implement such procedures. As modern pain control in the ED evolves, there are other blocks to explore in the hip, such as pericapsular nerve blocks,^13^ and elsewhere in the body such as erector spinae blocks for visceral truncal pain or brachial plexus. We did find that the established safety profile and familiar anatomy of the fascia iliaca block function as a stepping stone to grow comfortable with regional anesthesia and explore more opportunities to better manage our patients’ pain.

When developing a FICB program, using the ethics committee and having a QI process is important. Every ultrasound-guided FICB was reviewed, with feedback given to the performing clinician via our usual departmental point-of-care ultrasound quality assurance (QA) processes at our institution. Including the QA/QI component of care assists the implementation and ongoing operations related to delivering FICBs in the ED.

## LIMITATIONS

A variety of factors potentially limit the success of this implementation. This process was implemented at a tertiary trauma center with in-house access to all necessary specialties. Centers that see fewer high acuity patients may not receive patients with hip fractures or may transfer them to higher levels of care. This can make having the numbers needed for physician credentialing a challenge. This was also performed in a higher resource setting, limiting generalizability to other environments such as a community hospital with fewer resources and no learners. The landmark-based approach does offer a potentially more feasible option in some of these environments, including but not limited to time, staff, and equipment requirements.

## CONCLUSION

Our experience shows that within approximately one year of implementation it is possible to significantly increase utilization of the fascia iliaca compartment block in ED patients with a documented hip fracture. Our capture of 61% of eligible patients in this period is notably higher than previously documented implementation protocols. We believe the fundamental components of successful implementation at our institution included multidisciplinary collaboration, ED leadership support, a core group of emergency physicians leading the FICB program, material and educational support for the training process that provides spaced repetition in training and communication, and hospital-specific QA/QI processes that bolster continuation of the implemented protocols. Due to the success of this project, we have expanded our program to freestanding EDs and have started to use this as a platform for other nerve blocks and associated research.
